# Comparison of lumbar muscle morphology in patients with chronic nonspecific low back pain with and without clinical lumbar segmental instability

**DOI:** 10.1371/journal.pone.0301726

**Published:** 2024-04-04

**Authors:** Faranak Mahmoudi Alami, Mohammad Taghipour, Ghadamali Talebi, Payam Sa’adat, Tahere Seyedhoseinpoor, Hamid Vahidi Rad, Sorayya Khafri

**Affiliations:** 1 Faculty of Rehabilitation Medicine, Department of Physiotherapy, Babol University of Medical Sciences, Babol, Iran; 2 Health Research Institute, Mobility Impairment Research Center, Babol University of Medical Sciences, Babol, Iran; 3 Faculty of Medicine, Department of Radiology, Mazandaran University of Medical Sciences, Sari, Iran; 4 Faculty of Health, Department of Biostatistics and Epidemiology, Babol University of Medical Sciences, Babol, Iran; King Khalid University, SAUDI ARABIA

## Abstract

**Objectives:**

Evaluation of spinal muscle morphology may be critical because of its impact on segmental stability and control of the lumbar spine in the subset of patients with clinical lumbar segmental instability (LSI). The purpose of this study was to compare lumbar muscle morphology in CNLBP patients with clinical LSI, CNLBP patients without clinical LSI.

**Methods:**

This case-control study included 30 patients with CNLBP (15 with clinical LSI and 15 without clinical LSI) and 15 subjects without LBP. Axial magnetic resonance images from the L2 to S1 lumbar levels were used to evaluate the morphology of the lumbar muscles.

**Results:**

A significant increase in the muscle-to-fat infiltration index and a significant decrease in the relative muscle cross-sectional area (rmCSA) of the multifidus muscle at the L3-L4 to L5-S1 levels were observed in both CNLBP groups compared to the control group (p<0.05). The mean erector spinae mean rmCSA was significantly greater in the clinical LSI group compared to the control group (SMD = 0.853, 95% CI = 0.105 to -1.6, P = 0.044) and also compared to the CNLBP without clinical LSI (SMD = 0.894, 95% CI = -1.645 to -0.144, P = 0.030) at the L4-L5 level.

**Conclusions:**

The atrophic changes of the multifidus muscle, in CNLBP patients with or without clinical LSI was observed. However, hypertrophic changes of the erector spinae muscle at the L4-L5 lumbar level were observed only in the clinical LSI group. Psaos major did not show significant atrophic or hypertrophic changes.

## Introduction

Low back pain (LBP) is one of the most common musculoskeletal disorders, and its prevalence is increasing daily due to aging and population growth [1]. There are several causes of LBP, but in 90% of the cases there is no specific pathology, which is referred to as non-specific LBP [2]. Clinical lumbar segmental instability (LSI) is a type of chronic non-specific LBP (CNLBP). It accounts for 30–35% of the cases of CNLBP [3]. Lumbar segmental instability is defined as a significant reduction in the ability of the spinal stabilizing system to maintain the neutral zone within the physiologic range so that there is no neurologic dysfunction, major deformity, or disabling pain [3,4].

The lumbar muscles, such as the multifidus (MF), erector spinae (ES), and psoas major (PM), play an important role in maintaining lumbar segmental stability and controlling intervertebral motion. The MF, an important stabilizer of the lumbar neutral zone, provides two-thirds of active spinal stability. The ES helps maintain static trunk balance by resisting flexion moments imposed by gravity and loads anterior to the spine, and the PM works in concert with the posterior structures to dynamically maintain posture and stabilize the spine [5–7]. Inadequate muscle support may be one of the critical factors in the development and persistence of LBP. Lumbar muscle imaging may provide a means to assess the integrity of specific lumbar muscles at different spinal levels [8]. Although morphologic information of the muscle can be obtained by magnetic resonance imaging (MRI) or computed tomography (CT) and ultrasound imaging techniques, however, MRI is preferable to other methods for the following reasons: high contrast of soft tissues, no risk of ionizing radiation compared to CT scan, often prescribed in standard clinical care for cases of persistent LBP lasting more than three months, provides precise and reliable measurements [9–11].

Changes in lumbar muscle morphology have been reported in various pathologic conditions of the lumbar spine, such as degenerative disc disease or lumbar disc herniation, facet joint osteoarthritis, spondylolisthesis, lumbar spinal stenosis, and lumbosacral radiculopathy [12–17]. Lee et al. reported small functional cross-sectional area of the MF with high degree of fat infiltration and large functional cross-sectional area of the ES in the degenerative lumbar spondylolisthesis patient with chronic radiculopathy [15]. A systematic review that was recently conducted by Seyedhoseinpoor et al. showed that people with LBP have smaller MF muscles with a significant amount of intramuscular fat infiltration [18]. And meaningful associations between morphologic changes of the MF and ES with greater intervertebral motion have been reported in people with chronic LBP in a recent exploratory study [19]. Although the role of lumbar muscles in maintaining stability has been emphasized as an active subsystem of stability; but probable morphological change of lumbar muscle has not been clearly defined by literature in CLBP patients with clinical LSI. Besides, we know that clinical LSI contains prevalent subgroup of CLBP patients whose pain seems to be more due to muscular dysfunction. Therefore, the aim of the present study was to compare the morphology of the lumbar muscles such as MF, ES, and PM in CNLBP patients with signs and symptoms of clinical LSI and without clinical instability and also with those without LBP.

## Method

The sample size for this case-control study was determined using Gpower software according to the study by D’Hooge et al [9], considering a 4-point difference for muscle-to-fat infiltration indices with a confidence level of 95% and a power of 80%, and was calculated to be 15 for each group of patients and then 15 healthy subjects as a control group. Fifteen patients with CNLBP (9 females and 6 males) with aged 18 to 45 years with symptoms and signs of clinical LSI and fifteen patients with CNLBP without clinical LSI (7 females and 8 males) participated. Fifteen individuals with no history of LBP (7 females and 8 males) also participated as a control group.

Eligible subjects who were referred to a public hospital physiotherapy clinic between December 7, 2020 and May 24, 2021 were invited to participate in the study. All the patients were evaluated by spine specialist for diagnosis of CNLBP who had experienced persistent LBP or recurrent pain for at least three months, and at least six months had elapsed since their first episode of LBP. After checking the exclusion criteria of the study, they were invited to participate in the study if they were eligible. Exclusion criteria included pregnancy, vertebral fracture, herniated disc, acute back pain, systemic diseases, osteoarthritis, spondylolisthesis, LBP caused by trauma, nerve involvement such as tingling, numbness, and sharp pain, history of spinal surgery, inability to perform MRI due to obesity, or any specific cause leading to LBP [20]. Then, according to the criteria for assessing segmental instability, which was checked by the first authors, they were assigned to one of the groups of patients with or without clinical LSI.

Study groups: a) Patients with clinical LSI: patients with CNLBP <45 years of age with a negative straight leg raising (SLR) test, at least one positive aberrant movement pattern (such as a painful arc in lumbar flexion or when returning from flexion to upright standing, the Gowers sign, the instability catch, and a disturbed lumbopelvic rhythm) and a positive prone instability test at the lower level of the lumbar vertebrae [21,22]; b) Patients without clinical LSI: Patients with CNLBP without clinical symptoms of LSI as described for the previous group; c) Subjects without LBP: Subjects without LBP were also recruited by local announcement, and it was tried to include the healthy subjects without any notable history of LBP lasting more than three months for at least one year [23], with the age range close to the previous groups and a normal MRI report. Note that none of the study samples were athletes or specifically trained athletes.

This study was approved by the Ethical Review Board of Babol University of Medical Sciences (Ethical Code: IR.MUBABOL.REC.1399.340), and was conducted in accordance with the principles of the Declaration of Helsinki. Written informed consent was obtained from all subjects after explanation of the study objectives and prior to enrollment. After recording anthropometric information including height and weight for calculating body mass index (body mass index = weight/ height^2^), patients’ pain intensity and disability status were assessed respectfully using visual analog scale (VAS) and Oswestry Disability Index (ODI) questionnaires. Participants then underwent lumbosacral magnetic resonance imaging using a 1.5 Tesla scanner (General Electric, USA). Imaging was performed in a symmetrical supine position with the patient’s knees on a pillow. The following settings were used for magnetic resonance images: 4 mm slice thickness with 5 mm spacing; field of view 320×320 mm^3^, TR = 238 ms, TE = 113.4 ms, matrix size 192×256, flip angle of 90 degrees.

**Fig 1:**
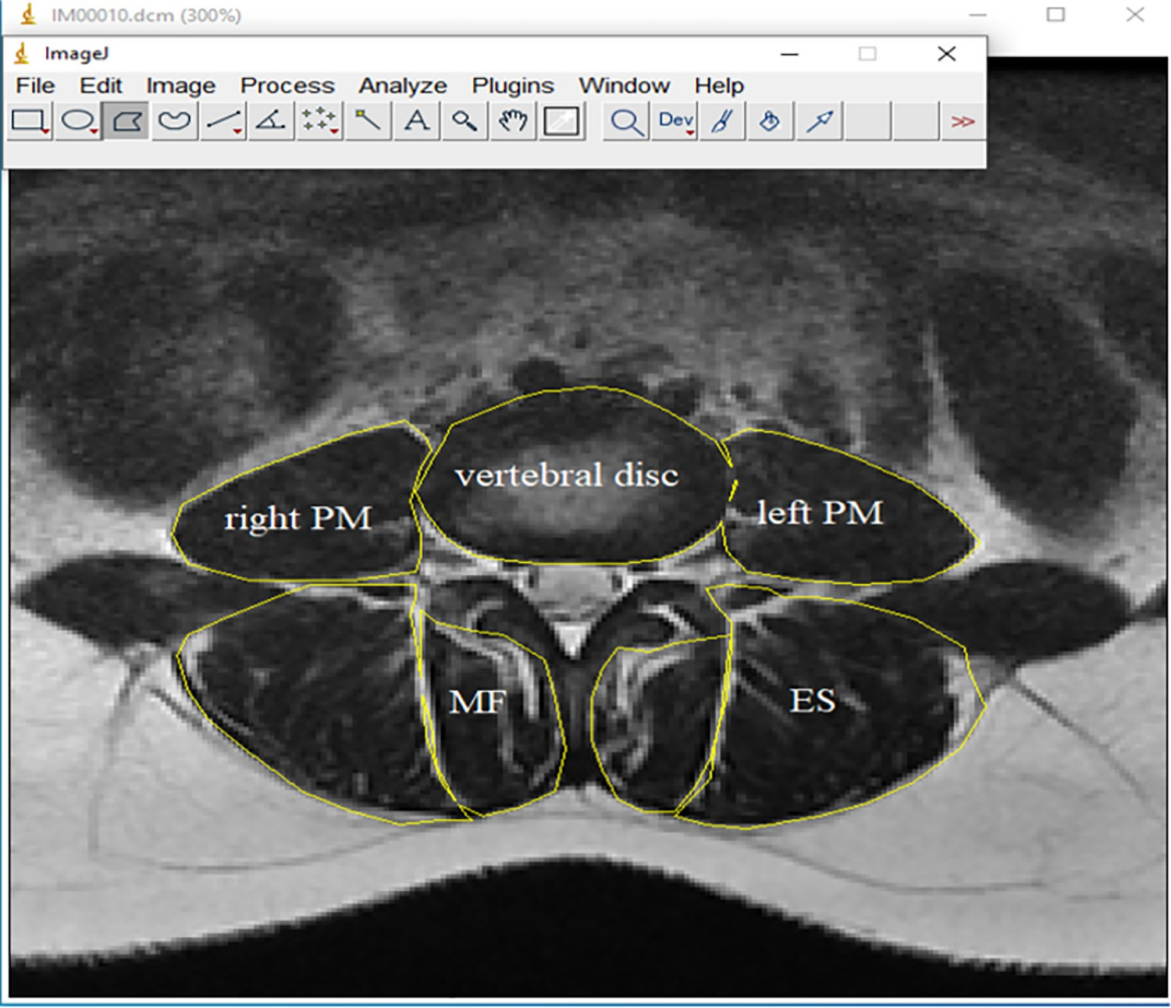
MRI of the lumbar spine in axial view at L4–L5 lumbar level.

On the axial T2 weighted images from L2 to S1, the cross-sectional area (CSA) of the right and left MF, ES, and PM muscles was measured by tracing within the fascial lines using the polygon tool of ImageJ software (National Institutes of Health; version 1.4.3.67) (Fig 1) [24]. The measured cross-sectional area of the muscle was divided by the cross-sectional area of the disc at that level to adjust for the effect of the patient’s height, weight, and body shape [12]. Using the histogram function of the software, summaries of the mean pixel intensity, which contained muscle and intramuscular fat, were obtained for the total CSA for each muscle at the corresponding vertebral levels. Fat pixel intensity summaries were determined for 0.5×0.5 cm^2^ areas of extramuscular fat lateral to the ES (to compare MF and ES) and lateral to the PM (to compare PM). Muscle-to-fat infiltration indices (MFIs), a measure of MRI-visible intramuscular fat, were calculated by dividing the mean CSA of fat pixel intensity by the mean pixel intensity of extra-muscular fat for each muscle. The relative cross-sectional area of the muscle (rmCSA) was also calculated using the formula (1 –MFI) × rCSA, to remove the fat portion of the muscle from the CSA. [6,24]. Right and left measurements were averaged for each level. Since the cross-sectional area of the muscles has been found to be related to the muscle’s ability to produce force, and it has been proposed that an increase in intramuscular fat negatively affects muscle contractility [15], muscle CSA, mCSA and MFI were extracted from axial images, which were considered important morphological parameters.

An experienced radiologist with more than 10 years of experience, blinded to the study sample group, performs all measurements. To check the reliability of the measurements, the MRI images of the L3-L4 level of a random sample of 15 participants were measured twice with an interval of two weeks.

Statistical analyses were performed using SPSS software version 26. Means and standard deviations were used for descriptive statistics of the variables. Intraclass correlation coefficient (ICC) and standard error of measurement (SEM) were used to assess the relative and absolute reliabilities. The normality assumption was checked and appropriate transformations were performed when necessary. One-way ANOVA was used to analyze the quantitative variables in the three groups. Tukey’s post hoc test was used for pairwise comparisons and p-values less than 0.05 were considered statistically significant. The standardized mean difference was used as a measure of the effect size to show the magnitude of the difference between the two groups; while the interval between 0.2 and 0.5 indicates a small effect size, the interval between 0.5 and 0.8 indicates a medium effect size, and more than 0.8 indicates a large effect size.

## Results

### Characteristics of participants

Forty-five subjects were allocated into 3 groups of CNLPB patients with clinical LSI, CNLBP patients without clinical LSI and individuals without LBP. Table 1 represented each group characteristics (Study dataset in S1 Appendix).

**Table 1:** Study sample characteristics by groups.

Group Variable	without LBP (n = 15)	CNLBP with Clinical LSI (n = 15)	CNLBP without Clinical LSI (n = 15)	p-value
Sex (female n, %)	9, 60%	7, 46.66%	7, 46.66%	0.701
Age (y)	34.13±4.92	34.4±7.33	38.4±6.16	0.122
Body mass index (kg/m^2^)	26.06±1.93	26.99±1.3	27.01±1.25	0.166
Pain intensity (cm)	-	4.7±1.68	4.8±2.43	0.823
ODI	-	31.67±9.00	30.53±12.38	0.777

LBP; Low Back Pain, CNLBP; Chronic Nonspecific Low Back Pain, LSI; Lumbar Segmental Instability, ODI; Oswestry Disability Index.

### LBP and MF morphology

**Fig 2:**
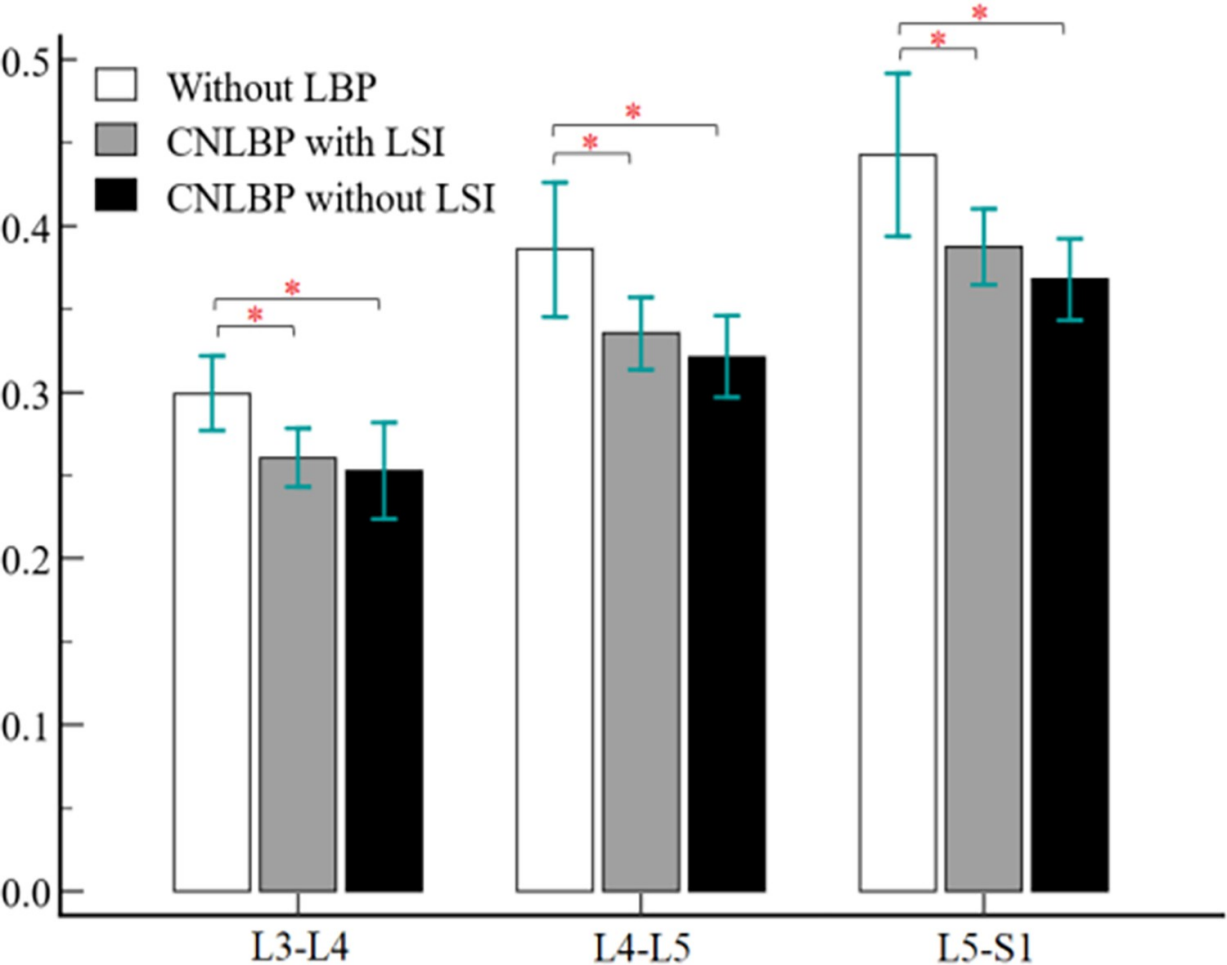
Comparison of multifidus rmCSA among the three groups.

As shown in Table 2, rCSA of MF muscle did not show statistically significant difference from L2-L3 to L5-S1 level among the studied groups. Meanwhile, rmCSA of MF muscle was significantly different between groups in all lumbar levels except L2-L3 level (L3-L4: F = 5.240, η2 = 0.200, P = 0.009, L4-L5: F = 5.904, η2 = 0.219, P = 0.006, L5-S1: F = 5.861, η2 = 0.218, P = 0.006). According to the pairwise comparisons, the mean rmCSA was significantly smaller in both CNLBP groups with clinical LSI with large effect size (SMD = -1.09, 95% CI = -1.857 to -0.323, P = 0.042) and without clinical LSI with large effect size (SMD = -1.013, 95% CI = -1.773 to -0.253, P = 0.011) compared to the control group without LBP at the L3-L4 level (Table 3). At the L4-L5 and L5-S1 levels, significantly lower mean rmCSA was also observed in both CNLBP groups compared to the control groups without LBP. All of the results that were statistically significant have a large SMD. (Table 3 for more details). However, the mean rmCSA did not show a significant difference between the CNLBP groups with clinical LSI and without clinical LSI at the above-mentioned levels (Fig 2).

**Table 2:** Multifidus, erector spinae, and psoas major muscle parameters and analysis of variance results.

			Studied groups						p-value	F	Eta Squared
			Without LBP		CNLBP with Clinical LSI		CNLBP without Clinical LSI				
			Mean	SD	Mean	SD	Mean	SD			
**Multifidus**	L2-L3	rCSA	0.30	0.05	0.28	0.04	0.29	0.05	0.610	0.500	0.023
		rmCSA	0.22	0.04	0.21	0.03	0.21	0.05	0.806	0.216	0.010
		MFI	0.26	0.05	0.24	0.04	0.27	0.06	0.327	1.149	0.052
	L3-L4	rCSA	0.40	0.05	0.37	0.04	0.36	0.06	0.088	2.581	0.109
		rmCSA	0.29	0.04	0.26	0.03	0.25	0.05	0.009*	5.240	0.200
		MFI	0.26	0.04	0.30	0.03	0.30	0.05	0.008*	5.441	0.206
	L4-L5	rCSA	0.53	0.08	0.51	0.05	0.48	0.06	0.139	2.072	0.090
		rmCSA	0.38	0.07	0.33	0.03	0.32	0.04	0.006*	5.904	0.219
		MFI	0.29	0.05	0.34	0.02	0.33	0.04	0.003*	6.824	0.245
	L5-S1	rCSA	0.65	0.12	0.61	0.05	0.58	0.05	0.067	2.893	0.121
		rmCSA	0.44	0.08	0.38	0.04	0.36	0.04	0.006*	5.861	0.218
		MFI	0.33	0.03	0.37	0.02	0.37	0.05	0.016*	4.571	0.179
**Erector Spinae**	L2-L3	rCSA	1.17	0.14	1.20	0.15	1.16	0.11	0.759	0.277	0.013
		rmCSA	0.81	0.24	0.92	0.13	0.84	0.13	0.263	1.378	0.062
		MFI	0.24	0.03	0.23	0.03	0.26	0.05	0.208	1.632	0.072
	L3-L4	rCSA	0.96	0.08	0.98	0.15	0.95	0.08	0.789	0.239	0.011
		rmCSA	0.71	0.08	0.74	0.12	0.68	0.07	0.280	1.311	0.059
		MFI	0.25	0.04	0.24	0.03	0.28	0.05	0.083	2.641	0.112
	L4-L5	rCSA	0.77	0.07	0.84	0.09	0.77	0.05	0.055	3.111	0.129
		rmCSA	0.54	0.08	0.61	0.08	0.53	0.05	0.018*	4.415	0.174
		MFI	0.29	0.07	0.27	0.03	0.30	0.04	0.069	2.850	0.119
	L5-S1	rCSA	0.65	0.01	0.64	0.07	0.58	0.06	0.078	2.706	0.114
		rmCSA	0.42	0.09	0.44	0.06	0.38	0.07	0.105	2.375	0.102
		MFI	0.36	0.06	0.31	0.03	0.35	0.06	0.061	2.987	0.125
**Psoas Major**	L2-L3	rCSA	0.46	0.15	0.43	0.11	0.42	0.08	0.645	0.444	0.021
		rmCSA	0.35	0.12	0.34	0.08	0.32	0.07	0.695	0.367	0.017
		MFI	0.23	0.02	0.19	0.05	0.23	0.05	0.085	2.612	0.111
	L3-L4	rCSA	0.64	0.16	0.58	0.13	0.54	0.09	0.172	1.837	0.080
		rmCSA	0.50	0.13	0.47	0.09	0.42	0.08	0.141	2.053	0.089
		MFI	0.22	0.04	0.19	0.04	0.23	0.05	0.103	2.396	0.102
	L4-L5	rCSA	0.82	0.22	0.77	0.17	0.70	0.09	0.158	1.931	0.084
		rmCSA	0.64	0.17	0.61	0.13	0.53	0.08	0.080	2.677	0.113
		MFI	0.22	0.04	0.20	0.05	0.24	0.04	0.119	2.242	0.096
	L5-S1	rCSA	0.81	0.22	0.76	0.11	0.74	0.15	0.517	0.671	0.031
		rmCSA	0.61	0.16	0.59	0.09	0.55	0.11	0.469	0.771	0.035
		MFI	0.24	0.04	0.22	0.02	0.25	0.03	0.059	3.039	0.126

LBP; Low Back Pain, CNLBP; Chronic Nonspecific Low Back Pain, LSI; Lumbar Segmental Instability, rCSA; relative Cross-Sectional Area, rmCSA; relative muscle Cross-Sectional Area, MFI; Muscle-to-Fat Infiltration Index, SD; Standard deviation

*p<0.05.

**Table 3:** Pairwise comparison of muscle parameters in different groups.

			Without LBP & CNLBP with Clinical LSI			Without LBP & CNLBP without Clinical LSI			CNLBP with Clinical LSI & CNLBP without Clinical LSI		
			MD	SMD (CI)	p-value	MD	SMD (CI)	p-value	MD	SMD (CI)	p-value
**Multifidus**	L2-L3	rCSA	0.018 (-0.018 to 0.55)	-0.371 (-1.093 to 0.351)	0.594	0.006 (-0.036 to 0.048)	-0.107 (-0.823 to 0.609)	0.947	-0.012 (-0.050 to .024)	0.242 (-0.477 to 0.96)	0.785
		rmCSA	0.010 (-0.021 to 0.041)	-0.246 (-0.965 to 0.472)	0.818	0.008 (-0.029 to 0.046)	-0.16 (-0.887 to 0.557)	0.860	-0.001 (-0.033 to .030)	0.048 (-0.668 to 0.764)	0.996
		MFI	0.024 (-0.013 to 0.062)	-0.492 (-1.219 to 0.234)	0.468	-0.004 (-0.052 to 0.042)	0.079 (-0.637 to 0.795)	0.973	-0.029 (-0.070 to0.012)	0.54 (-0.188 to 1.269)	0.345
	L3-L4	rCSA	0.034 (0.001 to 0.066)	-0.788 (-1.531 to -0.045)	0.179	0.039 (-0.003 to 0.082)	-0.697 (-1.434 to 0.041)	0.103	0.005 (-0.033 to .044)	-0.116 (-0.833 to 0.6)	0.957
		rmCSA	0.038 (0.011 to 0.065)	-1.09 (-1.857 to -0.323)	0.042*	0.046 (0.011 to 0.081)	-1.013 (-1.773 to -0.253)	0.011*	0.008 (-0.024 to 0.040)	-0.187 (-0.904 to 0.53)	0.863
		MFI	-0.037 (-0.065 to -0.009)	1 (0.241 to 1.759)	0.034*	-0.044 (-0.074 to -0.013)	1.099 (0.331 to 1.866)	0.011*	-0.006 (-0.036 to 0.023)	0.179 (-0.538 to 0.896)	0.889
	L4-L5	rCSA	0.018 (-0.034 to 0.070)	-0.261 (-0.979 to 0.458)	0.751	0.050 (-0.005 to 0.105)	-0.68 (-1.416 to 0.056)	0.122	0.032 (-0.012 to 0.076)	-0.545 (-1.273 to 0.184)	0.411
		rmCSA	0.050 (0.006 to 0.094)	-0.881 (-1.63 to -0.131)	0.037*	0.064 (0.019 to 0.109)	-1.089 (-1.856 to-0.322)	0.006*	0.014 (-0.017 to 0.045)	-0.337 (-1.057–0.384)	0.761
		MFI	-0.053 (-0.083 to -0.023)	1.351 (0.558 to 2.145)	0.003*	-0.039 (-0.074 to -0.004)	0.845(0.098 to1.592)	0.031*	0.014 (-0.012 to 0.040)	-0.409 (-1.132 to 0.314)	0.621
	L5-S1	rCSA	0.044(-0.029 to 0.119)	-0.454 (-1.179 to 0.27)	0.353	0.076 (0.002 to 0.150)	-0.782(-1.525 to -0.04)	0.054	0.032(-0.011 to 0.075)	-0.552(-1.281 to 0.178)	0.581
		rmCSA	0.055 (0.003 to 0.107)	-0.805 (-1.549 to -0.061)	0.048*	0.074 (0.022 to 0.127)	-1.064 (-1.828 to -0.299)	0.006*	0.019 (-0.012 to 0.051)	-0.452 (-1.177 to 0.273)	0.672
		MFI	-0.039 (-0.063 to -0.015)	1.245 (0.463 to 2.027)	0.026*	-0.036 (-0.071 to -0.002)	0.783 (0.041 to 1.526)	0.040*	0.002 (-0.027 to 0.032)	-0.076 (-0.792 to 0.64)	0.982
**Erector Spinae**	L2-L3	rCSA	-0.029 (-0.114 to 0.086)	0.189 (-0.528 to 0.906)	0.841	0.007 (-0.092 to 0.107)	-0.06(-0.776 to 0.565)	0.989	0.036 (-0.067 to 0.141)	-0.266 (-0.985 to 0.453)	0.763
		rmCSA	-0.104 (-0.250 to 0.041)	0.539 (-0.189 to 1.268)	0.251	-0.030 (-0.176 to 0.115)	0.159 (-0.558 to 0.876)	0.884	0.074 (-0.027 to 0.175)	-0.546 (-1.275 to 0.183)	0.494
		MFI	0.008 (-0.018 to 0.034)	-0.228 (-0.946 to 0.49)	0.877	-0.020 (-0.056 to 0.015)	0.418 (-0.305 to 1.142)	0.424	-0.028 (-0.065 to 0.008)	0.569 (-0.161 to 1.299)	0.199
	L3-L4	rCSA	-0.022 (-0.115 to 0.071)	0.177 (-0.54 to 0.894)	0.855	0.004 (-0.060 to 0.069)	-0.058 (-0.774 to 0.658)	0.993	0.026 (-0.065 to 0.118)	-0.219 (-0.937 to 0.499)	0.795
		rmCSA	-0.026 (-0.106 to 0.054)	0.244 (-0.475 to 0.962)	0.750	0.032 (-0.029 to 0.093)	-0.392 (-1.115 to 0.33)	0.648	0.058 (-0.019 to 0.135)	-0.562 (-1.292 to 0.168)	0.250
		MFI	0.010 (-0.019 to 0.040)	-0.252 (-0.97 to 0.467)	0.786	-0.025 (-0.061 to 0.010)	0.546 (-0.183 to 1.275)	0.268	-0.036 (-0.068 to -0.003)	0.823 (0.078 to 1.568)	0.077
	L4-L5	rCSA	-0.062 (-0.128 to 0.002)	0.715 (-0.023 to 1.454)	0.095	0.001 (-0.042 to 0.045)	-0.035 (-0.75 to 0.681)	0.999	0.064 (-0.004 to 0.132)	-0.709 (-1.447 to 0.029)	0.086
		rmCSA	-0.069 (-0.130 to -0.008)	0.853(0.105 to 1.6)	0.044*	0.004 (-0.041 to 0.051)	-0.076(-0.792 to 0.64)	0.985	0.074 (0.011 to 0.136)	-0.894(-1.645 to -0.144)	0.030*
		MFI	0.025 (-0.005 to 0.055)	-0.625 (-1.358 to 0.108)	0.203	-0.008 (-0.040 to 0.024)	0.185 (-0.532 to 0.902)	0.848	-0.033 (-0.059 to -0.007)	0.964 (-0.208 to 1.72)	0.069
	L5-S1	rCSA	0.011 (-0.059 to 0.081)	-0.117 (-0.834 to 0.599)	0.932	0.068 (0.000 to 0.136)	-0.761 (-1.503 to -0.02)	0.088	0.057 (0.002 to 0.112)	-0.793 (-1.537 to -0.05)	0.178
		rmCSA	-0.022 (-0.081 to 0.036)	0.294 (-0.426 to 1.013)	0.702	0.038 (-0.024 to 0.100)	-0.461 (-1.186 to 0.264)	0.376	0.060 (0.009 to 0.112)	-0.895 (-1.645 to -0.144)	0.091
		MFI	0.046 (0.007 to 0.084)	-0.895 (-1.645 to -0.144)	0.073	0.006 (-0.040 to 0.053)	-0.113 (-0.829 to 0.603)	0.943	-0.039 (-0.078 to 0.000)	0.758 (0.017 to 1.499)	0.142
**Psoas Major**	L2-L3	rCSA	0.028 (-0.070 to 0.126)	-0.213 (-0.931 to 0.505)	0.793	0.039 (-0.051 to 0.130)	-0.323 (-1.043 to 0.398)	0.634	0.011 (-0.061 to 0.084)	-0.113 (-0.83 to 0.603)	0.962
		rmCSA	0.010 (-0.069 to 0.089)	-0.095 (-0.811 to 0.621)	0.958	0.030 (-0.046 to 0.106)	-0.293 (-1.013 to 0.427)	0.679	0.020 (-0.041 to 0.081)	-0.244 (-0.962 to 0.475)	0.841
		MFI	0.036 (-0.001 to 0.070)	-0.799 (-1.543 to -0.056)	0.117	0.002 (-0.029 to 0.033)	-0.049 (-0.764 to 0.667)	0.993	0.034 (-0.076 to 0.008)	0.606 (-0.126 to 1.338)	0.145
	L3-L4	rCSA	0.055 (-0.057 to 0.167)	-0.367 (-1.089 to 0.355)	0.506	0.094 (-0.007 to 0.195)	-0.695 (-1.431 to 0.042)	0.149	0.038 (-0.048 to 0.126)	-0.335 (-1.056 to 0.386)	0.715
		rmCSA	0.030 (-0.057 to 0.119)	-0.264 (-0.983 to 0.455)	0.719	0.079 (-0.006 to 0.164)	-0.695 (-1.431 to 0.042)	0.122	0.048 (-0.018 to 0.115)	-0.639 (-1.373 to 0.094)	0.441
		MFI	0.026 (-0.006 to 0.060)	-0.612 (-1.344 to 0.12)	0.273	-0.009 (-0.043 to 0.025)	0.197 (-0.521 to 0.914)	0.849	-0.036 (-0.072 to 0.000)	0.742 (-0.002 to 1.482)	0.100
	L4-L5	rCSA	0.049 (-0.102 to 0.200)	-0.46 (-1.185 to 0.265)	0.719	0.124 (-0.005 to 0.253)	0.327 (-0.394 to 1.047)	0.137	0.074 (-0.030 to 0.179)	0.78 (-0.037 to 1.522)	0.474
		rmCSA	0.023 (-0.095 to 0.141)	-0.146 (-0.863 to 0.571)	0.890	0.111 (0.007 to 0.214)	-0.807 (-1.551 to 0.063)	0.084	0.088 (0.000 to 0.175)	-0.759 (-1.5 to -0.018)	0.205
		MFI	0.023 (-0.014 to 0.061)	-0.46 (-1.185 to 0.265)	0.420	-0.015 (-0.052 to 0.021)	0.327 (-0.394 to 1.047)	0.684	-0.038 (-0.076 to 0.000)	0.78 (-0.037 to 1.522)	0.101
	L5-S1	rCSA	0.048 (-0.082 to 0.180)	-0.279 (-0.998 to 0.44)	0.710	0.048 (-0.072 to 0.210)	-0.371 (-1.093 to 0.351)	0.502	0.020 (-0.080 to 0.121)	-0.156 (-0.873 to 0.56)	0.940
		rmCSA	0.017 (-0.083 to 0.118)	-0.126 (-0.843 to 0.59)	0.929	0.057 (-0.051 to 0.166)	-0.394 (-1.116 to 0.329)	0.453	0.040 (-0.038 to 0.118)	-0.383 (-1.105 to 0.339)	0.678
		MFI	0.022 (-0.004 to 0.050)	-0.604 (-1.336 to 0.127)	0.218	-0.009 (-0.040 to 0.021)	-0.245 (-0.473 to 0.964)	0.765	-0.032 (-0.055 to -0.008)	1.033 (-0.271 to 1.794)	0.054

LBP; Low Back Pain, CNLBP; Chronic Nonspecific Low Back Pain, LSI; Lumbar Segmental Instability, rCSA; relative Cross-Sectional Area, rmCSA; relative muscle Cross-Sectional Area, MFI; Muscle-to-Fat Infiltration Index, SD; Standard deviation, MD; Mean Difference, SMD; Standardized Mean Difference, CI; Confidence Interval

*p<0.05.

**Fig 3:**
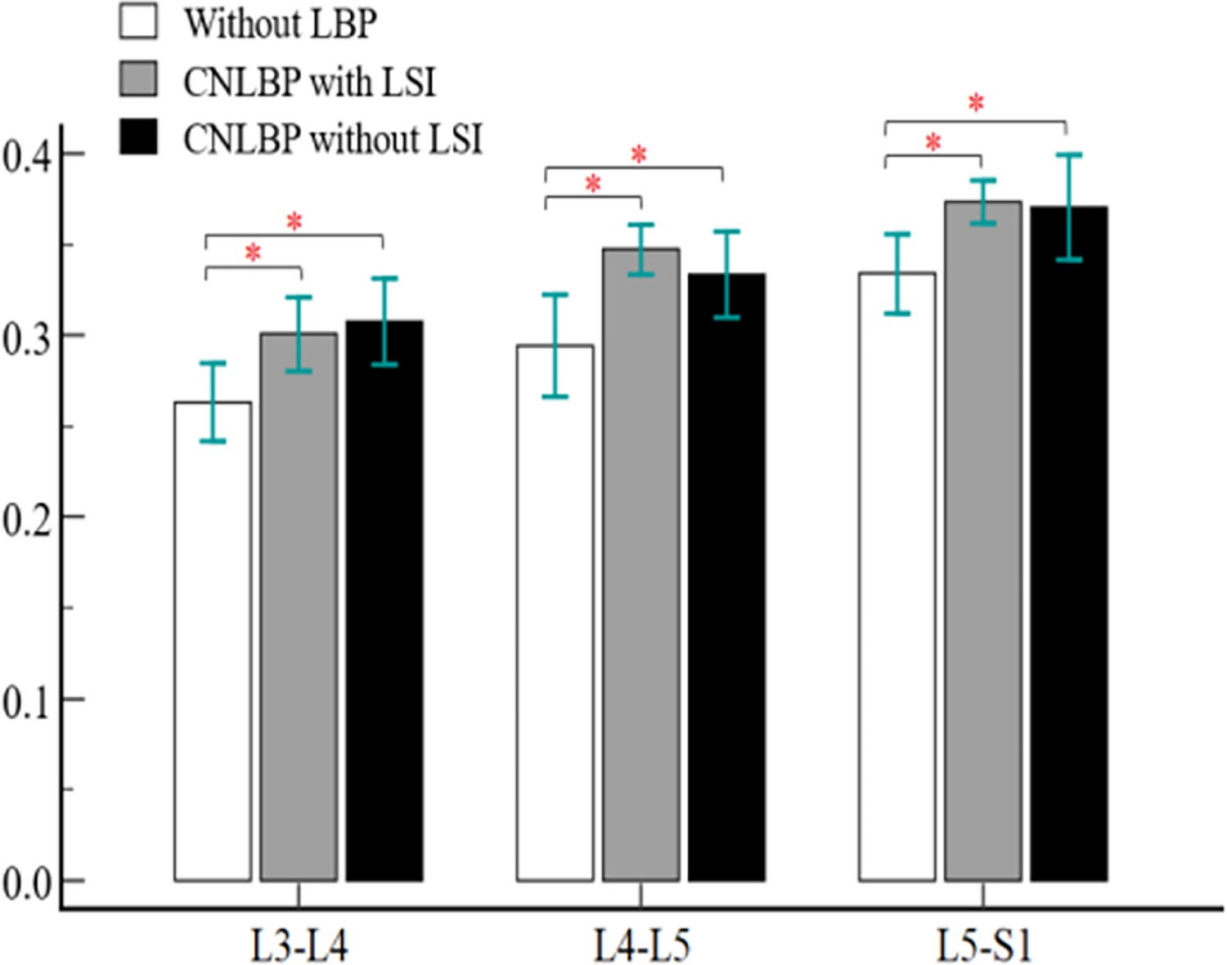
Comparison of multifidus MFI index among the three groups.

No statistically significant difference was found for MF MFI at L2-L3 level. But a significant difference was observed between the studied groups from L3-L4 to L5-S1 levels for MF FMI parameter. (L3-L4: F = 5.441, η2 = 0.206, P = 0.008, L4-L5: F = 6.824, η2 = 0.245, P = 0.003, L5-S1: F = 4.571, η2 = 0.179 P = 0.016). Pairwise comparisons at the L3-L4 level represented a significantly smaller MF MFI in the CLBP group with LSI compared to the control group without LBP with large effect size (SMD = 1, 95% CI = 0.241 to 1.759, P = 0.034) as well as in the CNLBP group without clinical LSI patients with large effect size (SMD = 1.099, 95% CI = 0.331 to 1.866, P = 0.011). Similar results were obtained for MF MFI at L4-L5 and L5-S1 levels. Compared with the control group without LBP, MF MFI was significantly lower in the clinical LSI group with large effect size (SMD = 1.351, 95% CI = 0.558 to 2.145, P = 0.003) and in the CNLBP without clinical LSI group with large effect size (SMD = 0.845, 95% CI = 0.098 to1.592, P = 0.031) at the L4-L5 level. And also, at L5-S1 level, CNLBP with clinical LSI group with large effect size (SMD = 1.245, 95% CI = 0.463 to 2.027, P = 0.026) and CNLBP without clinical LSI group with medium effect size (SMD = 0.783, 95% CI = 0.041 to 1.526 P = 0.040) had a smaller MF MFI. However, MF MFI did not show a statistically significant difference between CNLBP groups with and without clinical LSI at the levels studied (Fig 3).

### LBP and ES morphology

**Fig 4:**
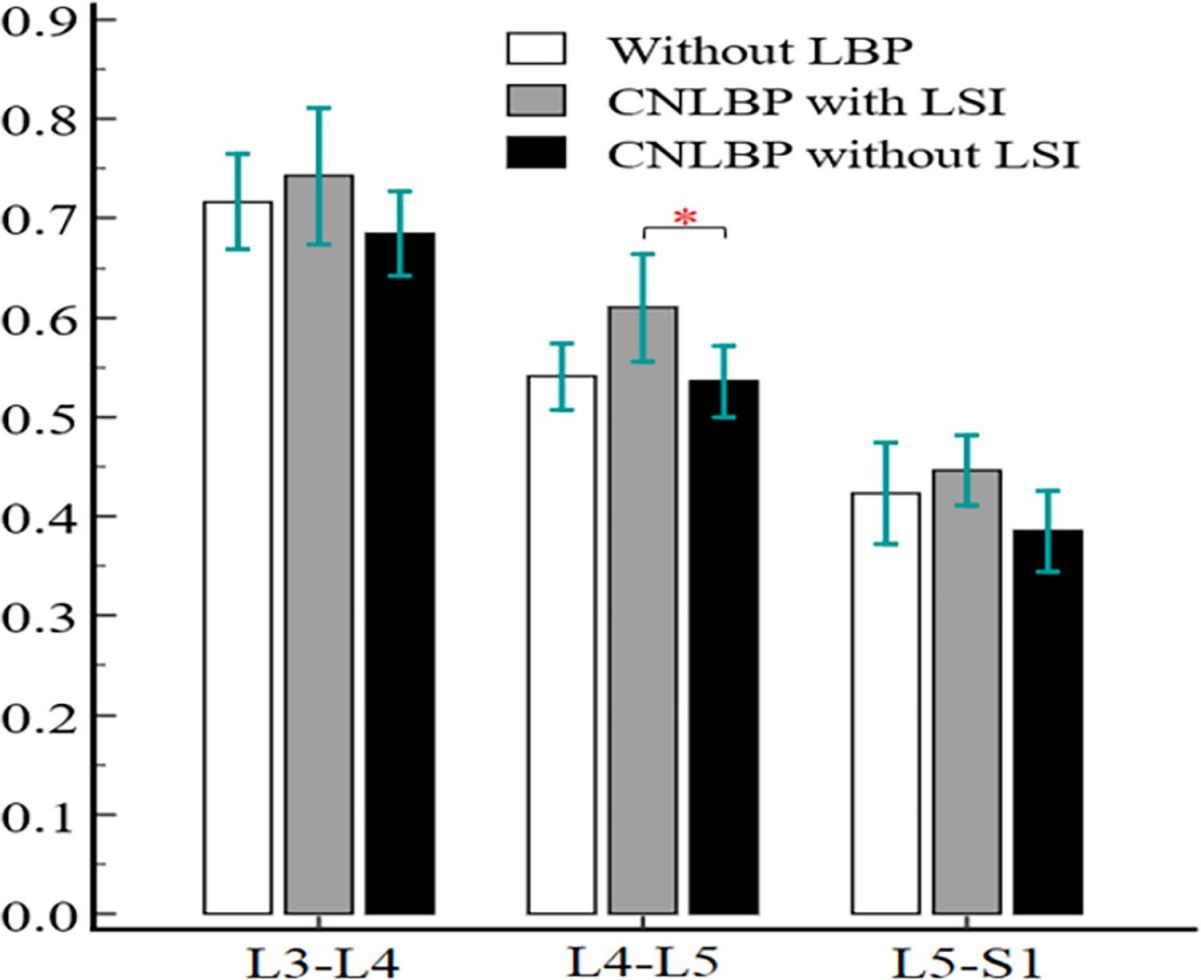
Comparison of erector spinae rmCSA among the three groups.

As illustrated in Table 2, among the investigated morphological parameters of the ES muscle, only the mean rmCSA at the L4-L5 level showed a statistically significant difference (F = 4.415, η2 = 0.174, P = 0.018). The mean rmCSA was significantly greater in patients with clinical LSI compared to the control group without LBP with large effect size (SMD = 0.853, 95% CI = 0.105 to 1.6, P = 0.044) and also compared to the CNLBP without clinical LSI group with large effect size (SMD = -0.894, 95% CI = -1.645 to -0.144, P = 0.030). However, the mean rmCSA was not significantly different between the CNLBP group without clinical LSI groups and the control group without LBP at this level (Fig 4). Mean rCSA and MFI did not show any statistically significant difference at this level, similar to the other evels (Table 3).

### LBP and PM morphology

None of the examined morphological parameters of the PM muscle showed a significant difference between the groups at any of the examined levels (Tables 2 and 3).

### Reliability

The intra-examiner intraclass correlation coefficient for estimating the variables of rCSA, MFIs and rmCSA of ​​the MF, ES and PM muscles at the level of the L3-L4 vertebrae ranged from 0.87 to 0.90 for the MF muscle, 0.88 to 0.96 for the ES muscle and 0.96 to 0.99 for the PM muscle, indicating excelent reliability of the measures.

## Discussion

This study showed that lumbar MF muscle atrophied with both decreased muscle size and increased muscle fat infiltration at L3-L4 to L5-S1 in CNLBP with or without clinical signs of LSI. In addition, the lumbar ES was hypertrophied at a single lumbar level (L4-L5 level) only in CNLBP patients with LSI. The PM muscle did not show any morphological changes in the CNLBP patients, either in those with LSI or in those without LSI.

Several studies have investigated the relationship between lumbar muscle morphology and different types of LBP. Consistent with the results of the present study, the changes in motor control, increasing MF fat infiltration and decreasing MF thickness were reported by Abdelaty et al. in patients with CNLBP with clinical lumbar instability [25]. Others such as Dayani et al. [26] found smaller MF size in CNLBP patients and Gobert et al. [27] found increased fat infiltration in the MF muscle with no change in total CSA in patients with CNLBP compared to patients with acute LBP. D’hooge et al. also reported the same in patients in the recovery phase of LBP [9]. A study by Yu using CT scan showed that in the lower lumbar region, the severity of fat infiltration in the MF muscle (and not the total CSA) was associated with facet joint osteoarthritis [17]. Systematic reviews have confirmed a moderate association between increased fat infiltration in the MF muscle and chronic LBP, but not for total CSA of this muscle in chronic LBP [18,27]. In a study by Lee et al, patients with degenerative lumbar spondylolisthesis had more fat infiltration in the MF muscle and low functional CSA [15]. Fat infiltration is a sign of muscle atrophy, and replacing muscle with fat may change muscle function but may not significantly change its CSA.

The residual effects of pain and dysfunction in different spinal muscles are different. Unlike the superficial muscles, which are mainly hyperactive, deep muscles such as the MF in the back and the transversus abdominis in the front (which are stabilizing muscles) are inhibited and their activity decreases. The MF consists of five myotomes, each of which receives a separate nerve from a specific spinal segment. Muscle fibers that attached to the spinous process of a particular vertebra are innervated by the medial branch of the dorsal ramus, which originates from the inferior to the corresponding vertebra [15]. According to the available evidence, the MF muscle plays a critical role in the lumbar segmental control. From a biomechanical point of view, the essential role of the MF is due to the action of the deep part of the muscle in controlling the intervertebral shear force. According to histological studies, the deep fibers of the MF muscle contain a higher percentage of type l fibers compared to other paraspinal muscles, and these slow-twitch fibers are more exposed to the adverse effects of pain and spinal immobility. This problem is one of the causes of the MF atrophy in the present study[15,19]. In this study, both groups of CNLBP with and without LSI showed almost similar MF atrophy. This finding confirms that MF atrophy is likely to be caused by pain and dysfunction regardless of the exact pathology [28].

The results of our study showed L4-L5 ES hypertrophy in patients with clinical LSIs, but not in other CNLBPs, which appears to be a compensatory response of an unstable spinal system. Consistent with our findings, a recent study reported a significant increase in erector spinae CSA in patients with positive structural LSI [19]. Previous studies of spondylolisthesis also showed hypertrophy of ES [15]. Lee studied the patients with chronic LBP patients with radiculopathy symptoms in two groups with or without spondylolisthesis and reported that erector spinae CSA increased in the spondylolisthesis, along with a decrease in multifidus CSA and an increase in fat infiltration of the muscle [1,15]. A study by Thakar showed that patients with spondylolisthesis had atrophy of the MF muscle and hypertrophy of the ES compared to healthy subjects. In a recent study of LBP, researchers acknowledged that hypertrophy of the ES muscle at the L4–L5 vertebral level may be due to a compensation or anatomical adaptation to the increased motion at the upper lumbar levels [19]. However, ES hypertrophy may have occurred as a compensatory response to poor stabilization of the lumbosacral spine due to pain, dysfunction, and deeper muscle atrophy. Furthermore, we didn’t find any hypertrophy of the ES in the CNLBP without LSI group. This may be due to the duration of CNLBP prior to evaluation, which we didn’t control for. In a recent study on structural LSI, researchers acknowledged that hypertrophy of the ES muscle at the L4–L5 vertebral level could be due to a compensation or anatomical adaptation to the increased motion at the upper lumbar levels [19], and there is no hypermobility at the vertebral segment in the CNLBP group without LSI, so the absence of hypertrophy of the ES muscle doesn’t seem unlikely.

The PM muscle has the highest CSA in the lower lumbar spine. Hip flexion is the primary function of this muscle. However, evidence suggests that this muscle acts as a stabilizer of the spine due to its high potential to generate compressive forces [13]. The present study did not demonstrate hypertrophic or atrophic changes in the PM muscle in relation to clinical LSI or other types of CNLBP. While studies have reported an association between the lower CSA of this muscle with aging and female gender [24], there is no evidence of an association between the morphology of this muscle and spondylolisthesis (as another type of lumbar instability with structural defects) [29]. One group of researchers believes that changes in the structure of the PM muscle follow structural changes in the vertebral column [30]. As we know, the increase in intervertebral motion is the problem in clinical lumbar segmental instability, not the structural changes in the vertebral column. Therefore, the absence of atrophic or hypertrophic changes in the PM associated with clinical LSI makes sense.

### Limitations

One of the advantages of this study was that it evaluated three parameters of muscle morphology for three lumbar muscles at four lumbar vertebral levels. Age, sex, BMI, and physical activity level are important covariates in the assessment of muscle morphology according to the literature [19]. The study sample was not large enough to allow analysis of covariates or the use of general linear models to determine their effects. However, age, sex, and BMI were not significantly different between groups, and none of the study samples participated in sports. Other limitations of the study were: although we know about the effects of medication and pain duration on muscle structure, the study was not able to control the patients’ medication as well as the exact duration of LBP. On the other hand, due to the observational nature of this study, we cannot establish causality, so studies with a stronger design such as cohort and randomized controlled trial are needed in the future to confirm the causality. In addition, further studies to investigate the effect of stabilizing exercises on lumbar muscle morphology and dynamic flexion-extension MRI to simultaneously investigate the clinical LSI and muscle morphology are recommended.

### Conclusions

Atrophic changes of the multifidus muscle, both in the form of a decrease in cross-sectional area and an increase in the muscle fat infiltration, were observed in patients with chronic nonspecific LBP with or without signs and symptoms of clinical lumbar segmental instability. However, hypertrophic changes of the ES muscle at the L4-L5 lumbar level were observed only in the patients with signs and symptoms of clinical lumbar segmental instability. The PM muscle did not show significant atrophic or hypertrophic changes in this study.

These results may pave the way for clinicians to pay more attention to increase the strength of the multifidus in general groups of nonspecific chronic low back pain. Moreover, to try to decrease the hyperactivity of the erector spinae muscles in groups of nonspecific chronic low back pain with signs and symptoms of clinical segmental instability. And the psoas seems to contribute less to nonspecific chronic low back pain, both with and without instability. More clinical research is needed to prove this.

## Supporting information

S1 Appendix Research dataset of the study. (XLSX)
